# [^124^I]IBETA: A New Aβ Plaque Positron Emission Tomography Imaging Agent for Alzheimer’s Disease

**DOI:** 10.3390/molecules27144552

**Published:** 2022-07-17

**Authors:** Grace A.H. Nguyen, Christopher Liang, Jogeshwar Mukherjee

**Affiliations:** Preclinical Imaging, Department of Radiological Sciences, University of California-Irvine, Irvine, CA 92697, USA; annn6@uci.edu (G.A.H.N.); liangc@uci.edu (C.L.)

**Keywords:** β-amyloid plaques, Alzheimer’s disease, imaging, transgenic 5 × FAD mice, postmortem human AD brain, iodine-124, iodine-125, autoradiography

## Abstract

Several fluorine-18-labeled PET β-amyloid (Aβ) plaque radiotracers for Alzheimer’s disease (AD) are in clinical use. However, no radioiodinated imaging agent for Aβ plaques has been successfully moved forward for either single-photon emission computed tomography (SPECT) or positron emission tomography (PET) imaging. Radioiodinated pyridyl benzofuran derivatives for the SPECT imaging of Aβ plaques using iodine-123 and iodine-125 are being pursued. In this study, we assess the iodine-124 radioiodinated pyridyl benzofuran derivative 5-(5-[^124^I]iodobenzofuran-2-yl)-N,N-dimethylpyridin-2-amine ([^124^I]IBETA) (Ki = 2.36 nM) for utilization in PET imaging for Aβ plaques. We report our findings on the radioiododestannylation reaction used to prepare [^124/125^I]IBETA and evaluate its binding to Aβ plaques in a 5 × FAD mouse model and postmortem human AD brain. Both [^125^I]IBETA and [^124^I]IBETA are produced in >25% radiochemical yield and >85% radiochemical purity. The in vitro binding of [^125^I]IBETA and [^124^I]IBETA in transgenic 5 × FAD mouse model for Aβ plaques was high in the frontal cortex, anterior cingulate, thalamus, and hippocampus, which are regions of high Aβ accumulation, with very little binding in the cerebellum (ratio of brain regions to cerebellum was >5). The in vitro binding of [^125^I]IBETA and [^124^I]IBETA in postmortem human AD brains was higher in gray matter containing Aβ plaques compared to white matter (ratio of gray to white matter was >5). Anti-Aβ immunostaining strongly correlated with [^124^/^125^I]IBETA regional binding in both the 5 × FAD mouse and postmortem AD human brains. The binding of [^124^/^125^I]IBETA in 5 × FAD mouse and postmortem human AD brains was displaced by the known Aβ plaque imaging agent, Flotaza. Preliminary PET/CT studies of [^124^I]IBETA in the 5 × FAD mouse model suggested [^124^I]IBETA was relatively stable in vivo with a greater localization of [^124^I]IBETA in the brain regions with a high concentration of Aβ plaques. Some deiodination was observed at later time points. Therefore, [^124^I]IBETA may potentially be a useful PET radioligand for Aβ plaques in brain studies.

## 1. Introduction

Alzheimer’s disease (AD) is the most common cause of dementia, which is frequently diagnosed in patients older than 60 years of age. It is a progressive neurodegenerative disorder that causes brain atrophy, neuronal death, and the loss of neuronal connections, leading to irreversible memory loss and cognitive impairment. In advanced stages of AD, complications from severe loss of brain function—such as dehydration, malnutrition, or infection—may be fatal. It has been challenging for clinicians to distinguish between the cognitive decline related to normal aging and that of AD. Currently, an absolute diagnosis of AD can only be determined by postmortem histopathological examination, although in vivo imaging is making major advances in pursuit of this diagnosis. Upon examining the postmortem brains of AD patients, the disease is shown to be associated with neuropathological features including the accumulation of amyloid β (Aβ) plaques, also known as senile plaques, and neurofibrillary tangles (NFT) of highly phosphorylated tau in the brain [1]. The deposition of Aβ plaques in the brain increases slowly and eventually plateaus in regions of early amyloid formation, such as the temporal associative isocortex, soon after the onset of the cognitive degeneration symptoms, or even during the preclinical stages of the disease, while NFT formation continues throughout the course of the disease [2]. Accordingly, Aβ plaque accumulation may start years before the onset of illness and even emerge well in advance of NFT formation [3]. The Aβ fragment, Aβ42, is shown to be dominant in Aβ plaques in AD brains [4], resulting from increased γ-secretase activity [5]. Additionally, mutations in the APP gene, which generally lead to an increase in Aβ peptide concentration, have been shown to cause some forms of early-onset familial AD, whereas mutations in tau genes without the presence of Aβ plaques can lead to non-Alzheimer dementias with neurofibrillary pathology [6]. Commonly used experimental animal models of AD are transgenic mice that overexpress human genes associated with familial AD (FAD) that lead to the formation of Aβ plaques. In particular, this study utilizes 5 × FAD transgenic mouse models, that contain three AD-linked mutations in human APP and two AD-linked mutations in PSEN1 genes: the Florida (I716V), Swedish (K670N/M671L), and London (V717I) mutations in APP; and the M146L and L286V mutations in PSEN1 [7].

**Figure 1:**
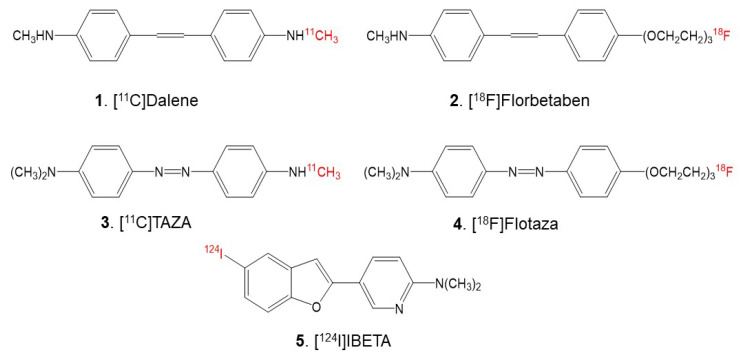
Chemical structures of Aβ plaque-binding PET radioligands. **1.** [^11^C]Dalene; **2.** [^18^F]Florbetaben; **3.** [^11^C]TAZA; **4.** [^18^F]Flotaza; **5.** [^124^I]IBETA.

Although the root cause of most Alzheimer’s cases still remains unknown, numerous findings based on genetic evidence signify a higher level of Aβ deposits in the brain, which correlates with a lower concentration in cerebrospinal fluid, which is essential in the pathogenesis of AD [8]. Therefore, imaging Aβ plaques in the brain may be helpful for AD risk prediction, the diagnosis of cerebral amyloidosis, and evaluating the efficacy of anti-amyloid treatments. Positron emission tomography (PET) studies of Aβ accumulation in AD have shown clinical utility [9]. Neuroimaging studies have conducted extensive research on the accumulation of Aβ plaques in the brains of AD patients: some further support previous pathological findings [1]. Successful clinical research trials utilizing [^11^C]PiB for diagnosing Aβ in AD patients and assessing the therapeutic efficacy of drugs for AD have facilitated the development and translation of PET for clinical use [10]. Clinical studies of [^11^C]PiB showed that it could detect Aβ deposition in AD patients and possibly identify persons with mild cognitive impairment [11,12]. The highly selective binding of [^11^C]PiB for insoluble amyloid plaques in the AD human brain was significantly correlated with post-mortem quantitative analyses [13]. However, the extensive clinical use of [^11^C]PiB is limited by the [^11^C] agent’s short half-life and the requirement of a cyclotron facility on site. Hence, several fluorine-18-labeled PET radiotracers are now being clinically used as they offer the advantages of a longer half-life than carbon-11-labeled agents. [^18^F]Florbetabir was the first fluorine-18 agent approved for clinical use, followed by [^18^F]Florbetaben (Figure 1) and [^18^F]Flutemetamol. 

Since the heteroatoms in the “azo” functionality of [^11^C]TAZA (Figure 1) result in a higher affinity to Aβ plaques and lower white matter binding in postmortem human AD brain slices in comparison to [^11^C]PiB, a fluorine-18 analog of [^11^C]TAZA—[^18^F]Flotaza (Figure 1)—has recently been developed and evaluated for Aβ plaque imaging [14,15]. [^18^F]Flotaza yielded significantly higher binding in the gray matter regions where Aβ plaques were located by anti-Aβ immunostains, compared to [^11^C]TAZA. Additionally, [^18^F]Flotaza showed a higher gray matter to white matter ratio than [^18^F]Florbetaben, a close fluoropegylated analog of [^11^C]Dalene (Figure 1), which exhibited a low amount of gray matter binding [15]. 

Single-photon emission computed tomography (SPECT) studies of Aβ plaque imaging have not advanced as much as PET studies [16]. Radioiodinated derivatives using iodine-125 and iodine-123 for imaging Aβ plaque has been pursued by several investigators [16,17,18,19]. Biodistribution studies and preliminary results in mouse models using iodine-123 pyridyl benzofuran and imidazo[1,2-a]pyridine derivatives have been reported [17,18]. Promising preliminary human SPECT imaging studies using [^123^I]ABC577 were reported and compared with known PET imaging agents [19]. However, further studies on the in vivo evaluation of the radiodinated SPECT agents in either transgenic mouse models or human AD have not been reported.

In our pursuit for a radioiodinated Aβ plaque imaging agent for in vivo and in vitro studies, we identified ^125^I-labeled 5-(5-Iodobenzofuran-2-yl)-N,N- dimethylpyridin-2-amine as a suitable candidate because of its high affinity [17]. Previous studies with this molecule included radiosynthesis using iodine-125 and normal mice biodistribution studies. Iodine-123 radiolabeling and in vivo SPECT imaging in transgenic mice were not carried out, although preliminary human AD brain slices in vitro exhibited promising properties [17]. Thus, our interest is in further evaluating the properties of this iodobenzofuran derivative. Deiodination has not been reported in previous studies, and it remains unclear if radio-deiodination is a concern with the in vivo imaging studies with these derivatives.

The goal of this study is to assess the feasibility of using 5-(5-[^124/125^I]iodobenzofuran-2-yl)-N,N-dimethylpyridin-2-amine (acronym: [^124/125^I]IBETA) (Figure 1) for Aβ plaque imaging. Iodine-124 (longer half-life of 4.2 days)-labeled PET imaging agents may be suitable for extended PET imaging. Additionally, since PET has a higher resolution compared to SPECT, it may be useful for the identification of small brain regions in transgenic AD mice brains with Aβ plaque accumulation. Thus, we report the following: (a) the radiosynthesis of [^124/125^I]IBETA; (b) the evaluation of drug effects on the binding of [^125^I]IBETA in a 5 × FAD mouse model known to have robust Aβ plaques [20] and postmortem human AD brain slices in vitro; (c) the evaluation of the binding of [^124^I]IBETA to Aβ plaque in postmortem human AD and 5 × FAD mouse brain slices in vitro; and (d) preliminary PET/CT studies of [^124^I]IBETA in a living 5 × FAD AD mouse model.

## 2. Materials and Methods

### 2.1. General Methods

All chemicals and solvents were purchased from Aldrich Chemical and Fisher Scientific. Deionized water was acquired from the Millipore Milli-Q Water Purification System. Iodine-125 sodium iodide, carrier free (specific activity = 643 MBq/µg) in 0.01 N NaOH, was purchased from American Radiolabeled Chemicals, Inc., St. Louis, MO, USA. Iodine-124 sodium iodide, carrier free (specific activity = >1000 GBq/µmole) in 0.01N NaOH, was purchased from 3D Imaging, LLC., Little Rock, AK, USA. Iodine-124 and iodine-125 radioactivity were counted in a Capintec CRC-15R dose calibrator while low level counting was carried out in a Capintec Caprac-R well-counter. All solvents used were provided by Fisher Scientific. Gilson high-performance liquid chromatography (HPLC) was used for semi-preparative reverse-phase column chromatography with a UV detector set at dual wavelengths of 254 nm and 280 nm as well as a radioactivity detector. A semi-preparative HPLC column of 100 × 250 mm and a 10-micron Econosil C18 reverse-phase was used. Analytical thin-layer chromatography (TLC) was used to monitor the reactions (Baker-flex, Phillipsburg, NJ, USA). Radio TLC was scanned on an AR-2000 imaging scanner (Eckart & Ziegler, Berlin, Germany). Electrospray mass spectra were obtained from a Model 7250 mass spectrometer (Micromass LCT). Proton NMR spectra were recorded on a Bruker OM EGA 500-MHz spectrometer. Brain slices were prepared at 10µm thick using the Lieca 1850 cryotome. In vitro-labeled brain sections were exposed to phosphor films (Perkin Elmer Multisensitive, Medium MS) and read using the Cyclone Phosphor Imaging System (Packard Instruments). Analysis of in vitro autoradiographs was performed using Optiquant acquisition and analysis software.

### 2.2. Animals

All animal studies were approved by the Institutional Animal Health Care and Use Committee of University of California, Irvine. IACUC PHS Assurance number, D16-00259 (A3416-01)

#### 2.2.1. C57BL/6 Mice

Adult mice were used in this study (28 g). Mice were purchased from Jackson Laboratory and housed under controlled temperatures of 22 °C ± 1 °C, in a 12 h light–dark cycle, on at 6:00 AM, with water and food chow ad libitum.

#### 2.2.2. 5 × FAD Transgenic Mice

The 5 × FAD transgenic line of mice (MMRRC hemizygous; 4 male and 4 female) were purchased from Jackson Laboratory. Female mice were 20–28 g and male mice weighed 26–38 g. All mice were housed in standard cages. 

### 2.3. Human Tissue

Human postmortem brain tissue samples were obtained from Banner Sun Health Research Institute, Sun City, AZ brain tissue repository for in vitro experiments. Age- and gender-matched AD brain and cognitively normal (CN) brain tissue samples were used for the study. Human postmortem brain slices were obtained from chunks of frozen tissue on a Leica 1850 cryotome cooled to −20 °C. Iodine-124 and Iodine-125 autoradiographic studies were carried out by exposing tissue samples on storage phosphor screens (Perkin Elmer Multisensitive, Medium MS and tritium sensitive phosphor screens). The apposed phosphor screens were read and analyzed by OptiQuant acquisition and the analysis program of the Cyclone Storage Phosphor System (Packard Instruments Co., Boston, MA, USA). Adjacent slices were used for immunostaining with anti-Aβ. All postmortem human brain studies were approved by the Institutional Biosafety Committee of the University of California, Irvine. 

### 2.4. Synthesis 

5-(5-Bromobenzofuran-2-yl)-N,N-dimethylpyridin-2-amine (Br-BETA) was synthesized using previously published methods [17]. 5-bromobenzofuran-2-boronic acid (72 mg, 0.299 mmol) was treated with 5-iodo-N,N-dimethylpyridin-2-amine (66 mg, 0.266 mmol) in the presence of (Ph_3_P)_4_Pd (36 mg, 0.0312 mmol) in 2M Na_2_CO_3_ (aq.)/dioxane (15 mL, 2:1). The reaction mixture was stirred under reflux overnight. The mixture was removed from the heat and allowed to cool to room temperature. Then, 1M NaOH (2 mL) was added to the mixture while being stirring at room temperature. The organic phase was extracted using ethyl acetate and was then dried over MgSO_4_. After filtration, the solvent was removed by vacuum rotary evaporation, and the residue was further purified using silica gel thin-layer chromatography (TLC) in hexane/ethyl acetate (4:1). The experimental yield was low (6.66%). Mass spectra (ESI): *m*/*z* 318 ([M + H]^+^, 100%).

**Figure 2:**
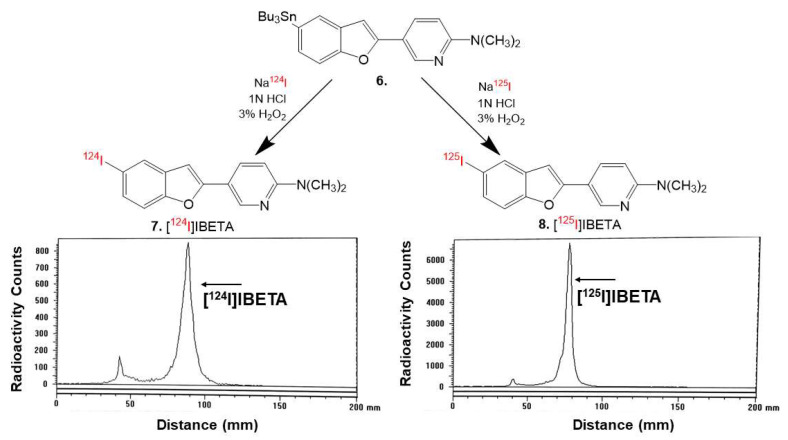
[^124^I]NaI and [^125^I]NaI were used to prepare [^124^I]IBETA **6** and [^125^I]IBETA **7** by electrophilic substitution of the tributyltin derivative. TLC of [^124^I]IBETA shows the purity of >85%, and TLC of [^125^I]IBETA shows the purity of >95%.

N,N-Dimethyl-5-(5-(tributylstannyl)benzofuran-2-yl)pyridin-2-amine (Figure 2) was synthesized based on previously published methods [17]. The bromobenzofuran intermediate (5.6 mg, 0.0177 mmol) was treated with bis(tributyltin) (0.0150 mL) in the presence of (Ph_3_P)_4_Pd (1.869 mg, 0.00162 mmol) in a dioxane/triethylamine solvent mixture (0.8 mL, 3:1). The mixture was stirred at 90 ^°^C overnight. The product was extracted using silica gel TLC in hexane/ethyl acetate (4:1) as the product traveled faster and had a higher R_f_ value than the starting material. The experimental yield was low (16.0%). Mass spectra (ESI): *m*/*z* 528 ([M + H]^+^, 100%).

### 2.5. Radiosynthesis

Sodium iodide, [^124^I]NaI (3D Imaging LLC) and [^125^I]NaI (ARC Inc.) were used to prepare [^124^I]IBETA and [^125^I]IBETA by the electrophilic substitution of the tributyltin derivative using reported radioiodination methods [21,22]. The radiosynthesis of [^124^I]IBETA using methods modified from [22] and [^125^I]IBETA using methods modified from [15,16] were successfully caried out. Then, 0.1 mL H_2_O_2_ (3%) was added to a mixture of 0.1 mL of tributyltin derivative (1 mg/0.2 mL of ethanol), 21 MBq NaI^124^, and 0.1 mL of 1N HCl in a sealed vial. The reaction was allowed to proceed at room temperature for 30 min before it was terminated by the addition of sodium bisulfite. The solvent was removed by vacuum rotary evaporation and [^124^I]IBETA was purified by HPLC. Radio TLC confirmed a radiochemical purity of >85% [^124^I]IBETA (Figure 2). Molar activity was estimated to be >500 TBq/mmole under the no-carrier added conditions

The same reaction was used to synthesize [^125^I]IBETA from the tributyltin derivative (1.5 mg/0.7 mL of ethanol) and 3.4 MBq [^125^I]NaI. The reaction was allowed to proceed at room temperature for 60 min before it was terminated by the addition of sodium bisulfite 0.1 M. The purification and isolation of [^125^I]IBETA were conducted on preparative TLC. Two rounds of extraction were performed using dichloromethane. The extract was then dried using anhydrous MgSO_4_. Radio TLC confirmed a radiochemical purity of >95% [^125^I]IBETA (Figure 2). Molar activity was estimated to be approximately 90 TBq/mmole under the no-carrier added conditions.

### 2.6. In Vitro Mice Brain Autoradiography

All experiments were carried out in accordance with the Institutional Animal Care and Use Committee at the University of California, Irvine, and were consistent with Federal guidelines. Male and female hemizygous 5 × FAD seven-month-old mice obtained from MMRRC JAX were used for in vitro and in vivo studies. Horizontal brain slices were sectioned (10 μm thickness) on a Leica 1850 Cryostat and collected on Fisher slides. The slides contained three to four brain sections each, were placed in separate glass chambers (six slides per chamber), and were preincubated in PBS buffer for 15 min. The preincubation buffer was discarded. The brain slices were treated with [^124^I]IBETA and [^125^I]IBETA in 40% ethanol PBS buffer pH 7.4 (60 mL, 5 kBq/mL). The chambers were incubated at 25 ^°^C for 1.25 h. The nonspecific binding of [^125^I]IBETA was measured in separate chambers using Flotaza and Br-BETA (0.1 µM). The slices were then washed with cold PBS buffer, 90% ethanolic PBS buffer twice, PBS buffer, and cold water. The brain sections were air-dried and exposed overnight on a phosphor film (Multisensitive Medium MS, PerkinElmer, Waltham, MA). The apposed phosphor screens were read and analyzed by OptiQuant acquisition and the Cyclone Storage Phosphor System (Packard Instruments Co., Boston, MA, USA). The regions of interest (ROIs) were drawn on the slices and the extent of binding of [^124^I]IBETA and [^125^I]IBETA was measured in DLU/mm^2^. 

### 2.7. In Vitro Postmortem Human Brain Autoradiography

All experiments were carried out in accordance with the Institutional Review Board at the University of California, Irvine, and were consistent with Federal guidelines. Human AD (*n* = 6) post-mortem brain tissues were obtained from Banner Health Research Institute, Sun City, Arizona. Brain slices were sectioned (10 μm thickness) on a Leica 1850 Cryostat and collected on Fisher slides. The slides contained three to four brain sections each were placed in separate glass chambers (six slides per chamber) and were preincubated in PBS buffer for 15 min. The preincubation buffer was discarded. The brain slices were treated with [^124^I]IBETA and [^125^I]IBETA in 40% ethanol PBS buffer pH 7.4 (60 mL, 5 kBq/mL). The chambers were incubated at 25 ^°^C for 1.25 h. Nonspecific binding was measured in separate chambers using Flotaza and Br-BETA (0.1 µM) in the presence of [^125^I]IBETA. The slices were then washed with cold PBS buffer, 90% ethanolic PBS buffer twice, PBS buffer, and cold water. The brain sections were air-dried and exposed overnight on a phosphor film (Multisensitive Medium MS, PerkinElmer, Waltham, MA, USA). The apposed phosphor screens were read and analyzed by OptiQuant acquisition and the Cyclone Storage Phosphor System (Packard Instruments Co., Boston, MA, USA). ROIs were drawn on the slices and the extent of binding of [^124^I]IBETA and [^125^I]IBETA was measured in DLU/mm^2^. 

### 2.8. Immunohistochemistry

The immunostaining of all brain sections was carried out by University of California, Irvine, pathology services using Ventana BenchMark Ultra protocols. To determine the localization of Aβ accumulation in the subject brains, neighboring slices of postmortem human AD and mouse brain slices were immunostained with anti-Aβ Biolegend 803015 (Biolegend, CA, USA), which is reactive to amino acid residue 1–16 of Aβ. Immunostained sections were scanned using the Ventana Roche instrumentation and the images were analyzed using QuPath software.

### 2.9. Mice PET and CT Scanning

Animals had free access to food and water during housing. All animals were fasted for 18–24 h prior to PET imaging. In preparation for the scans, the mice were induced into anesthesia with 3% isoflurane. Inveon preclinical dedicated PET (Siemen’s Inc) was used for the MicroPET studies, which has a resolution of 1.45 mm [23]. The Inveon PET and MM CT scanners were placed in the “docked mode” for combined PET/CT experiments (Siemens Medical Solutions, Knoxville, TN, USA). A Sigma Delta anesthetic vaporizer (DRE, Louisville, KY, USA) was used to induce and maintain anesthesia during injections and PET/CT acquisitions. 

[^124^I]IBETA was taken in 10% alcoholic sterile saline (0.9% NaCl injection, United States Pharmacopeia) for injections into mouse models for PET/CT studies. [^124^I]IBETA was injected retro-orbitally (0.9 MBq) under 2% isoflurane anesthesia. The mice underwent 15 min PET scans (90 min and 24 h post-injection) in a supine position accompanied by a 7 min CT scan for attenuation correction. The CT images were reconstructed with a cone beam algorithm (bilinear interpolation, Shepp-Logan filter) into 480 × 480 × 632 image arrays with a 206 μm pixel size. Following the reconstruction, the CT images were spatially transformed to match the PET images. In addition to being reconstructed into an image, the CT data were used for the attenuation correction of the PET images.

### 2.10. Image Analysis

All in vivo images were analyzed using Inveon Research Workplace (IRW) software (Siemens Medical Solutions, Knoxville, TN, USA) and PMOD Software (PMOD Technologies, Zurich, Switzerland). Whole-body PET/CT images were analyzed using the IRW software for [^124^I]IBETA uptake and any other CT anomalies in the whole-body images [24]. For additional brain quantitative analysis, brain images were also analyzed using PMOD, with PET images co-registered to a mouse brain MRI template [24,25]. The magnitude of [^124^I]IBETA in each volume of interest, VOI (in kBq/mL), was measured. Cerebellum was used as a reference region in order to calculate the ratio of the target brain regions to the reference region.

## 3. Results

### 3.1. Synthesis

Using the previously published procedures from Ono et al., Br-BETA was synthesized to be used to prepare the tributyltin precursor for radioiodination. A tributyltin substituent successfully substituted the bromine of Br-BETA in <10% yield, which is still sufficient to be used in the radiolabeling experiments.

Hydrogen peroxide (H_2_O_2_) was used as an oxidant in the radiolabeling with NaI^124^ and NaI^125^. At room temperature, the radiolabeling with [^124^I]NaI proceeded for 30 min while the radiolabeling with [^125^I]NaI was allowed to proceed for 60 min before being terminated. RadioTLC was used to monitor the reaction progress. The HPLC purification of [^124^I]IBETA resulted in a singular radioactive peak with a radiochemical yield of approximately 40% and a purity of >85%, as shown by radio TLC (Figure 2). After 30 min, radio TLC monitoring of the reaction with [^125^I]NaI, however, showed little product; hence, the reaction was allowed to proceed for an additional 30 min. The purification and isolation of [^125^I]IBETA using preparative TLC was efficient, with little loss on the silica. The isolated product showed a purity of >95%, as indicated by radio TLC with a radiochemical yield of approximately 27%. Other alternative methods to improve the radiochemical yields of [^124^I]IBETA and [^125^I]IBETA with high specific radioactivity for in vivo studies will be explored further. 

### 3.2. [^125^I]IBETA in 5 × FAD Mouse Model

**Figure 3:**
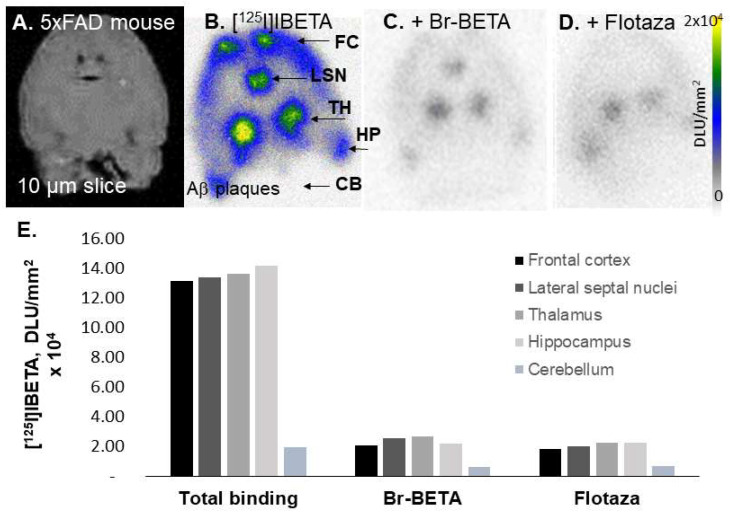
Competition of [^125^I]IBETA with drugs in 5 × FAD mouse model: (**A**) 5 × FAD mouse 10 µm brain slice; (**B**) high [^125^I]IBETA uptake in the thalamus (TH), frontal cortex (FC), lateral septal nuclei (LSN), and hippocampus (HP) in the autoradiographs; none in the cerebellum (CB); (**C**) Br-BETA (0.1 µM) effect on [^125^I]IBETA binding; (**D**) Flotaza (0.1 µM) effect on [^125^I]IBETA binding; (**E**) a plot comparing [^125^I]IBETA uptake in different brain regions with different drugs.

Male (*n* = 2) and female (*n* = 2) hemizygous 5 × FAD mouse brain slices were used to evaluate the total binding of [^125^I]IBETA, as well as the effect of Br-BETA and Flotaza on the Aβ binding of [^125^I]IBETA. All brain slices had a frontal cortex (FC), lateral septal nuclei (LSN), thalamus (TH), hippocampus (HP), and cerebellum (CB) (Figure 3A). In the absence of the other Aβ ligands, the in vitro binding of [^125^I]IBETA was significant in the TH, FC, and HP, while CB revealed very low levels of binding (Figure 3B). The ratio of regions to CB was approximately 7, suggesting high levels of Aβ plaques in these regions. Upon the addition of the bromo analog of I-BETA (Br-BETA, 0.1 µM), >85% of [^125^I]IBETA binding to Aβ plaques in the TH, FC, and HP were abolished, as expected, suggesting a high affinity for Aβ-binding sites (Figure 3C). Flotaza (0.1 µM), a high-affinity Aβ ligand, also displaced >85% of [^125^I]IBETA binding in the TH, FC, and HP, suggesting similar binding sites of [^125^I]IBETA and Flotaza (Figure 3D). The binding of [^125^I]IBETA in the FC, TH, and HP strongly correlated with anti-Aβ immunostains for Aβ plaques, hence confirming the binding of [^125^I]IBETA to the regions that contained high Aβ deposition (Figure 3E).

### 3.3. [^125^I]IBETA in Postmortem Human AD Brain

**Figure 4:**
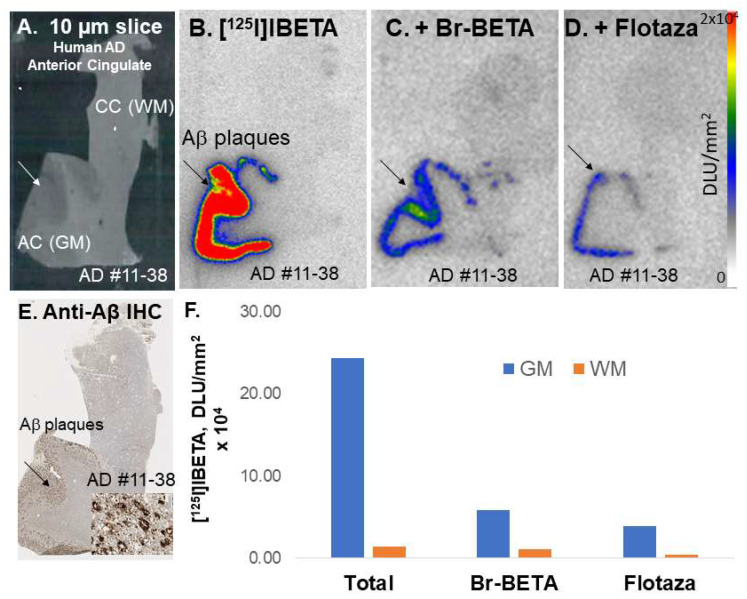
In vitro competition of [^125^I]BETA with drugs in human postmortem AD brains consisting of anterior cingulate. (**A**) Postmortem human AD 10 µm brain slice; (**B**) human postmortem AD brain shows [^125^I]BETA uptake mainly in the anterior cingulate (AC) in the autoradiographs but none in the corpus callosum (CC); (**C**) Br-BETA (0.1 µM) effect on [^125^I]BETA binding; (**D**) Flotaza (0.1 µM) effect on [^125^I]BETA binding; (**E**) anti-Aβ immunostains in AC, where most Aβ plaques are located; (**F**) a plot shows the displacement of Br-BETA and Flotaza by [^125^I]BETA in gray matter (GM) and white matter (WM) in the human AD brain.

Postmortem human AD subjects’ (*n* = 2) brain sections containing an anterior cingulate (AC) were used to evaluate the total binding affinity of [^125^I]IBETA to Aβ plaques (Figure 4A). In the absence of other Aβ ligands, the in vitro binding of [^125^I]IBETA was higher in the gray matter (GM) consisting of AC, while little binding was shown in the white matter (WM) comprising of corpus callosum (CC), and the AC to CC ratio was >20 (Figure 4B). Upon the addition of Br-BETA (0.1 µM), >80% of [^125^I]IBETA binding to Aβ plaques in the AC were displaced, suggesting a high affinity for Aβ-binding sites (Figure 4C). Flotaza (0.1 µM) displaced >80% of [^125^I]IBETA binding in the AC, indicating similar binding sites (Figure 4D). Additionally, [^125^I]IBETA binding in the AC strongly correlated with anti-Aβ immunostains for Aβ plaques in the same subjects, hence confirming the specific affinity of [^125^I]IBETA to Aβ plaques in the AD brain (Figure 4E).

**Figure 5:**
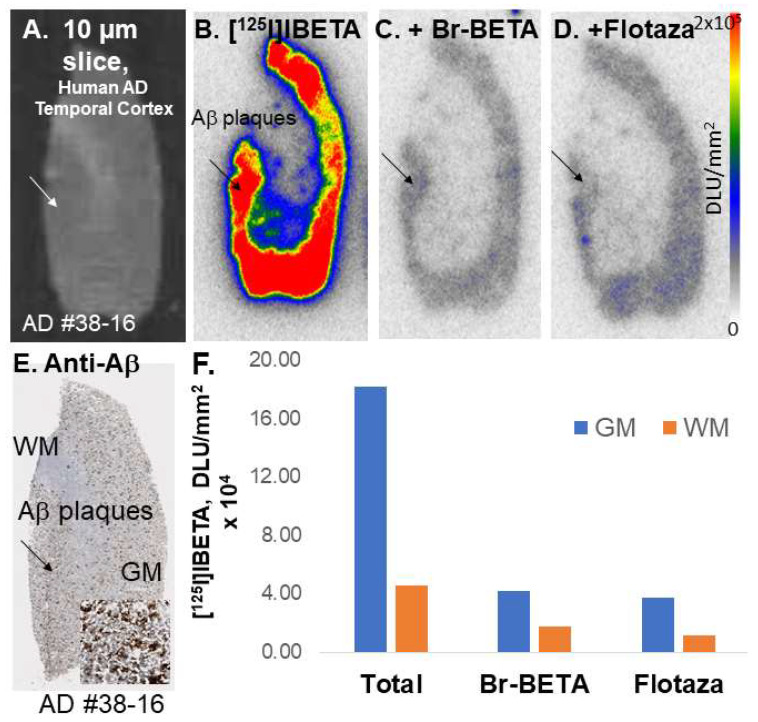
In vitro competition of [^125^I]BETA with drugs in postmortem human AD brains containing temporal cortex. (**A**) Postmortem human AD 10 µm brain slice; (**B**) human postmortem AD brain shows [^125^I]BETA uptake mainly in the gray matter (GM) in the autoradiographs but none in the white matter (WM); (**C**) Br-BETA (0.1 µM) effect on [^125^I]BETA binding; (**D**) Flotaza (0.1 µM) effect on [^125^I]BETA binding; (**E**) anti-Aβ immunostains in the AC, where most Aβ plaques are located; (**F**) a plot shows the displacement of Br-BETA and Flotaza by [^125^I]BETA in GM and WM in the human AD brain.

Temporal cortex from postmortem human AD subjects brains were also used to evaluate the total binding affinity of [^125^I]IBETA to Aβ plaques (Figure 5A). In the absence of other Aβ ligands, the in vitro binding of [^125^I]IBETA was higher in the gray matter, while little binding was shown in the white matter (WM) (Figure 5B). Upon the addition of Br-BETA (0.1 µM), >80% of [^125^I]IBETA binding to Aβ plaques were displaced, suggesting a high affinity for Aβ-binding sites (Figure 5C). Flotaza (0.1 µM) displaced >80% of [^125^I]IBETA binding, indicating similar binding sites (Figure 5D). Additionally, [^125^I]IBETA binding in the temporal cortex strongly correlated with anti-Aβ immunostains for Aβ plaques in the same subjects, hence confirming the specific affinity of [^125^I]IBETA to Aβ plaques in the AD brain (Figure 5E).

### 3.4. [^124^I]IBETA in 5 × FAD Mouse Model

**Figure 6:**
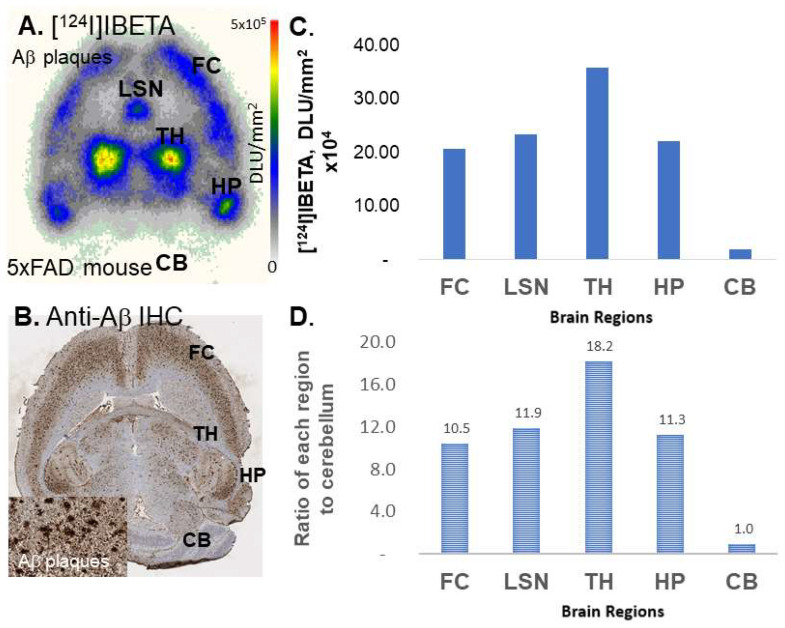
In vitro binding of ${[}^{124}$I]IBETA in 5 × FAD mouse brain model. (**A**) Uptake of ${[}^{124}$I]IBETA within the thalamus (TH), frontal cortex (FC), lateral septal nuclei (LSN), and hippocampus (HP) in autoradiographs; (**B**) anti-Aβ immunostains in 5 × FAD mouse brain where most Aβ plaques are located; (**C**) a plot shows the relative uptake of ${[}^{124}$I]IBETA in in vitro brain slices; (**D**) a plot shows the ratio of four brain regions in comparison to the cerebellum, which has little uptake.

Female hemizygous 5 × FAD (*n* = 3) seven-month-old mice were used to evaluate the total binding affinity of [^124^I]IBETA to Aβ plaques in these mouse brain sections. These were the same subjects used in the experiments with [^125^I]IBETA. All brain slices contained FC, LSN, TH, HP, and CB. The incubation of horizontal brain slices with [^124^I]IBETA showed significant binding in the TH, FC, and HP, while very low levels of binding were observed in the CB (Figure 6A). The ratio of regions to CB was >10, which was higher than the results of [^125^I]IBETA, suggesting high levels of Aβ plaques in these regions (Figure 6C,D). [^124^I]IBETA uptake in the FC, TH, and HP was highly consistent with anti-Aβ immunostains for Aβ plaques, hence confirming the binding of [^124^I]IBETA to the regions that contained high levels of Aβ plaque accumulation (Figure 6B).

### 3.5. [^124^I]IBETA in Postmortem Human AD Brain

**Figure 7:**
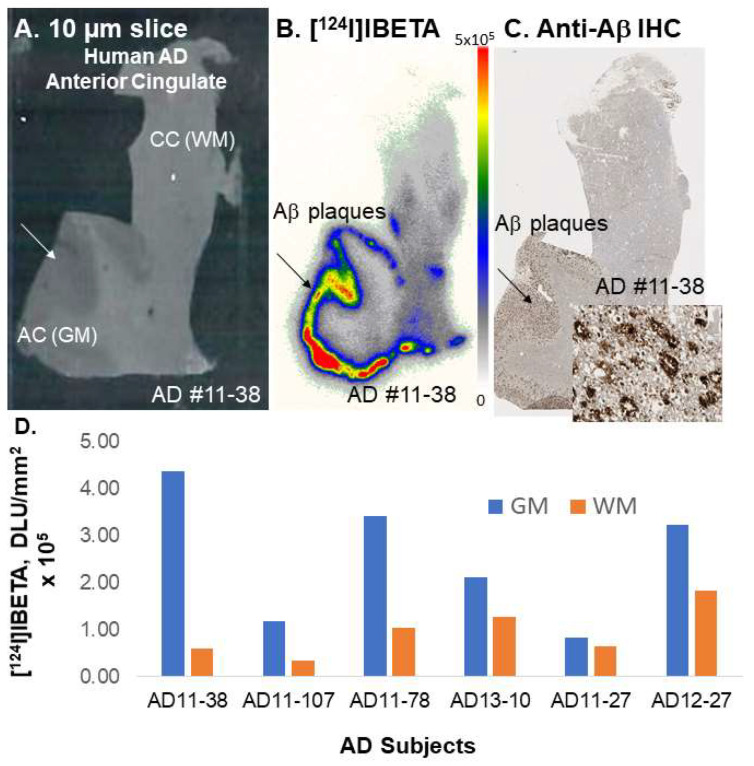
In vitro binding of [^124^I]IBETA in postmortem human AD brain. (**A**) Postmortem human AD 10 µm brain slice; (**B**) human postmortem AD brain shows [^124^I]IBETA uptake mainly in the anterior cingulate (AC) in the autoradiographs but none in the corpus callosum (CC); (**C**) anti-Aβ immunostains in AC, where most Aβ plaques are located; (**D**) a plot shows higher [^124^I]IBETA uptake in gray matter (GM) in the AC than in white matter (WM) in the CC in six AD subjects, suggesting higher levels of Aβ plaques present.

Postmortem human AD (*n* = 6) brain sections, in which AD11-38 and AD11-78 were also used in the experiments with [^125^I]IBETA, were used to evaluate the total binding affinity of [^124^I]IBETA to Aβ plaques. All brain sections contained WM consisting of the CC and GM consisting of the AC regions (Figure 7A). The in vitro binding of [^124^I]IBETA was higher in the AC, while little binding was shown in the CC (Figure 7B). The AC to CC ratio was >7 (Figure 7D). Very little WM binding was seen when alcohol was used for washing, while the WM binding increased significantly when washing was performed using only PBS. The binding of [^124^I]IBETA in the AC strongly correlated with anti-Aβ immunostains for Aβ plaques in the same subjects, confirming the binding of [^124^I]IBETA to Aβ plaques in the brain slice (Figure 7C).

### 3.6. Preliminary PET/CT Studies with [^124^I]IBETA

**Figure 8:**
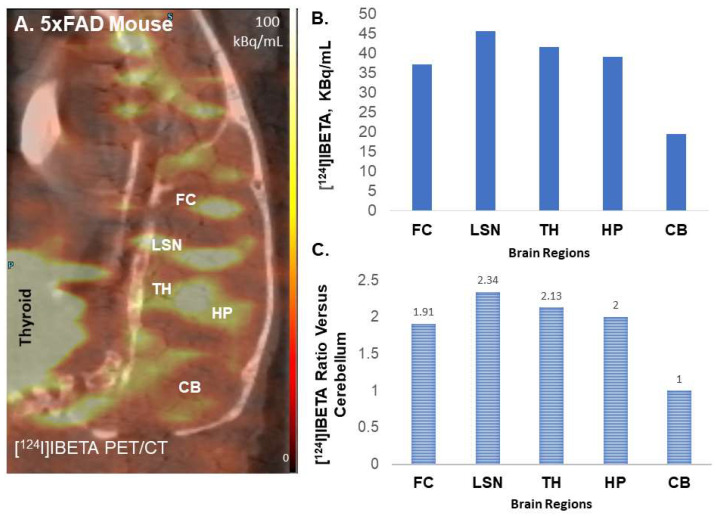
In vivo binding of [^124^I]IBETA in the 5 × FAD mouse model. (**A**) Summed PET/CT scan 70-90 mins post-[^124^I]IBETA administration showing retention of [^124^I]IBETA within the frontal cortex (FC), lateral septal nuclei (LSN), thalamus (TH), hippocampus (HP), and cerebellum (CB); (**B**) a plot shows the levels of [^124^I]IBETA uptake in different regions of the mouse brain; (**C**) a plot shows the ratio of four brain regions in comparison to the CB, which has little uptake.

To assess the feasibility of Aβ imaging in vivo, female hemizygous 5 × FAD (*n* = 3) seven-month-old mice were used for in vivo PET/CT [^124^I]IBETA studies. Previously reported studies with the radioiodinated analog [17] used the older Tg 2576 Aβ plaque model [26]. The uptake of [^124^I]IBETA in the brain was significant and localized in higher concentrations in the FC, LSN, TH and HP regions, while minimal uptake was shown in the CB (Figure 8A). This regional in vivo distribution of [^124^I]IBETA was consistent with in vitro experiments shown in Figure 6 for [^124^I]IBETA and in Figure 3 for [^125^I]IBETA. The ratio of different brain regions versus the cerebellum in vivo ranged from 1.9 to 2.3 (Figure 8C), whereas ratios of these brain regions versus the cerebellum in vitro were found to be much higher (Figure 6D). This may be expected due to the high nonspecific binding of [^124^I]IBETA in vivo. However, at later time points, [^124^I]IBETA cleared from the brain regions and a significant amount of activity was detected in the thyroid region, suggesting some deionization of [^124^I]IBETA in vivo. Minimal radioactivity was observed in most brain regions one-day post-injection with only <20% of the initial uptake remaining, indicating the efficient reversibility and washout of [^124^I]IBETA from the brain after 24 h of injection. Other PET radiotracers, such as [^11^C]PiB, have also been shown to exhibit the reversibility in binding to Aβ plaques.

**Figure 9:**
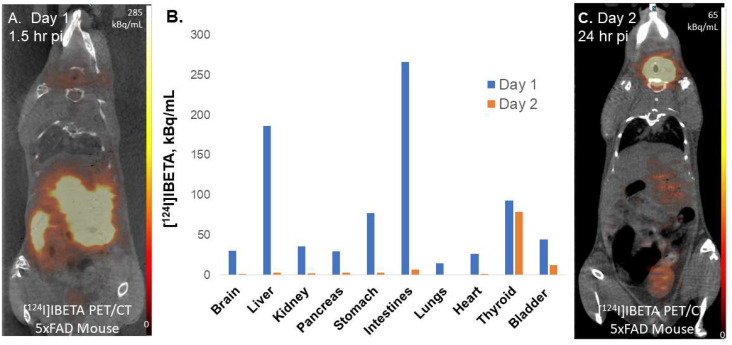
The biodistribution of radioactivity after injection of [^124^I]IBETA in 5 × FAD mouse model. (**A**) Uptake in different organs in PET/CT [^124^I]IBETA scan on the day of injection; (**B**) plot compares the levels of [^124^I]IBETA uptake in different organs 1.5 h post-injection (Day 1) and 24 h (Day 2) post-injection; (**C**) uptake in different organs in PET/CT [^124^I]IBETA scan one-day post-injection.

Moreover, we investigated the biodistribution of radioactivity to other organs throughout the body in addition to the brain after a retro-orbital injection of [^124^I]IBETA in the same mouse model. The liver, stomach, and intestines showed high initial uptake on day 1 of injection (Figure 9A). After one day post-injection, almost all of [^124^I]IBETA uptake was cleared from the body, with a higher rate of clearance in the liver, stomach, and intestines, which had significant initial uptake (Figure 9C). Nonetheless, a little amount of [^124^I]IBETA was cleared from the thyroid after one day of injection, as iodine typically participates in the synthesis of two main thyroid hormones, triiodothyronine (T3) and thyroxine (T4) (Figure 9B). The accumulation of radioactive iodine in thyroid is always the concern when iodinated radioligands are used. However, in clinical trials, the pre-administration of iodine-rich diet and fluids that contain stable iodine can competitively suppress the nonspecific absorption of radioiodine in normal tissues [27,28].

## 4. Discussion

Imaging biomarkers show much potential for illness diagnosis, disease progression monitoring, therapeutic response tracking, and advancing our current understanding of the physiology and pathology of AD. Fluorine-18 has been an ideal radionuclide due to its half-life of 110 min, which allows adequate time to radiolabel the drug of interest and localize it in vivo. Nonetheless, iodine-124 and iodine-125 have a much longer half-life than the traditional fluorine-18 radionuclide, which is often prepared from a cyclotron on-site due to its short half-life. The recent development of novel 123/125-iodinated pyridyl benzofuran Aβ radiotracers showed a high affinity for Aβ plaques in vitro [17]. For further investigation, our study examined the effects of different drugs on the binding of [^125^I]IBETA in vitro, which had not been reported in the previous publication. However, iodine-125 radiolabeled drugs are more commonly utilized in laboratory experiments and biodistribution studies due to their long half-life (60 days) and low energy level of emitting radiation. In the pursuit of a radioiodinated analog of [^123/125^I]IBETA, we evaluated [^124^I]IBETA as a possible candidate for Aβ imaging in vitro and in vivo, which can potentially be used in PET preclinical imaging for Aβ aggregates in human AD brain. This study is similar to our previous efforts on the extended imaging of dopamine receptors with iodine-124-labeled epidepride [22].

In our in vitro studies using 5 × FAD mouse brains, the bromo analog of I-BETA (Br-BETA) and Flotaza—an unlabeled fluorine analog of a new PET radiotracer for imaging Aβ plaques ([^18^F]Flotaza [15])—displaced >85% of [^125^I]IBETA binding, suggesting a good correspondence in the binding sites of these two Aβ ligands. Since the binding of [^125^I]IBETA was almost abolished completely in the presence of its bromo analog, a radiobrominated analog of Br-BETA, which can be labeled with ^75^Br (T_1/2_ = 97 min), ^76^Br (T_1/2_ = 16 h), or ^77^Br (T_1/2_ = 3.2 days), could be developed as potential PET radiotracer for Aβ plaques with longer physical half-life options [29]. In vitro studies using postmortem human AD brains, Br-BETA and Flotaza also displaced much [^125^I]IBETA binding (>80%), suggesting low non-specific binding in vitro. The strong correlation of [^125^I]IBETA uptake in vitro and anti-Aβ immunostains signifies the high affinity of this radioligand to Aβ plaques in AD brain. 

[^124^I]IBETA revealed a higher binding for Aβ plaques in the 5 × FAD mouse models, in comparison to its 125-iodinated analog, as shown by a higher ratio of regions to CB (>10). The higher specific activity of [^124^I]IBETA may lead to greater ratios compared to [^125^I]IBETA. The high uptake of [^124^I]IBETA in the TH and FC regions correlated well with anti-Aβ immunostains for Aβ plaques, hence confirming the binding of [^124^I]IBETA to regions that contained high Aβ accumulation. The uptake in the brain region in vivo PET/CT [^124^I]IBETA scans was consistent with in vitro binding in the 5 × FAD mouse brain slices, with high uptake in the TH and FC regions. According to the scans, [^124^I]IBETA successfully penetrated the blood–brain barrier upon injection and most radioactivity was cleared from the brain at 24 h post-injection. The biodistribution of radioactivity presents a similar behavior of [^124^I]IBETA in vivo compared to the previously reported distribution of [^125^I]IBETA [17]. 

A higher uptake of [^124^I]IBETA for Aβ plaques was observed in the GM than in the WM of postmortem human AD brain slices. The resulting AC to CC ratios were consistent with Aβ plaque localization in in vitro mouse brains. Nonetheless, one of the human AD subjects (AD11-38) showed much higher uptake in AC to CC (>8) in comparison to the other subjects. Thus, additional studies on the incubation condition for [^124^I]IBETA, as well as more subjects, might be necessary to address the discrepancy in [^124^I]IBETA binding in postmortem human AD brain slices. Furthermore, the binding of [^124^I]IBETA in brain slices containing AC and CC correlated well with the localization of Aβ aggregation consistent with this mouse model [20], which was also confirmed by anti-Aβ BioLegend 803015 immunohistochemical studies. 

## 5. Conclusions

In summary, [^124^I]IBETA is a new Aβ PET imaging agent, which can be made in one-step radiosynthesis followed by HPLC purification. It exhibited high binding to Aβ plaques in 5 × FAD mouse models and postmortem human AD brain tissues in vitro. These findings are consistent with the isotopic analog [^125^I]IBETA. The assessment of drug effects and the reversibility of the binding of [^124^I]IBETA on Aβ plaques in vitro has yet to be conducted. Nonetheless, since the extent of displacement of [^125^I]IBETA with Flotaza was >80% in both 5 × FAD mouse models and postmortem human AD brain slices, similar displacement patterns are expected for future experiments that use [^124^I]IBETA. The preliminary PET/CT scans and the mouse brain slice autoradiography of the 5 × FAD mice showed binding to Aβ plaques consistent with the 5 × FAD mice model. Hence, there is potential for [^124^I]IBETA to be used as a PET radiotracer to detect Aβ plaques for the evaluation of potential therapeutic agents [30] and comparison with other novel agents [31]. Furthermore, SPECT imaging studies on 5 × FAD mice using [^123^I]IBETA may be possible. Efforts to develop suitable SPECT imaging agents for Aβ plaques are being actively pursued [16,19].

## Data Availability

The data that support the findings of this study are available from the corresponding author upon reasonable request.
